# *Trichosanthes kirilowii* lectin alleviates diabetic nephropathy by inhibiting the LOX1/NF-κB/caspase-9 signaling pathway

**DOI:** 10.1042/BSR20180071

**Published:** 2018-09-07

**Authors:** Jiandong Lu, Jinting Peng, Min Xiang, Liangping He, Dongcai Wang, Guoliang Xiong, Shunmin Li

**Affiliations:** 1 Department of Nephrology, Shenzhen Traditional Chinese Medicine Hospital, Guangzhou University of Chinese Medicine, Shenzhen, Guangdong 518033, China; 2 Centers for Disease Early Treatment, Shenzhen Traditional Chinese Medicine Hospital, Guangzhou University of Chinese Medicine, Shenzhen, Guangdong 518033, China

**Keywords:** caspase-9, diabetic nephropathy, HK-2 cells, nuclear factor kappaB, Trichosanthes kirilowii lectin

## Abstract

*Trichosanthes kirilowii* lectin (TKL) has been reported to exert hypoglycemic effects in alloxan-induced diabetic mice. However, there is no evidence showing that it helps to prevent diabetic nephropathy (DN). We used a high glucose (HG)-induced HK-2 cell model and a streptozocin (STZ)-induced Wistar rat model to investigate the effects of TKL on DN, as well as the mechanisms for those effects. Our results showed that TKL significantly increased the viability of HG-treated HK-2 cells and inhibited cell apoptosis. *In vivo* experiments demonstrated that TKL attenuated STZ-induced histopathological damage and the inflammatory response in rat kidney tissues. Pre-treatment of HK-2 cells or STZ-treated rats with polyinosinic acid (Poly IC), an inhibitor of lectin-like oxLDL receptor 1 (LOX1), blocked the protective effect of TKL against HG- or STZ-induced damage to kidney tissue, indicating that TKL might exert its effect via LOX1-mediated endocytosis. Additional results suggested that TKL inhibits the phosphorylation of IκB kinase β (IKKβ) and the nuclear factor-κB (NF-κB) inhibitor protein (IκBα), and thereby reduces the nuclear translocation of NF-κB (p65). ChIP assay data indicated that TKL markedly inhibits the binding of p65 to the *CASP9* gene in HG-treated HK-2 cells, subsequently suppressing transcription of the *CASP9* gene. In the dual-luciferase reporter assay, TKL significantly inhibited luciferase activity in cells co-transfected with p65 and a wild-type capase-9 construct instead of mutated caspase-9 constructs.

Taken together, our results show that TKL helps to protect against DN by inhibiting the LOX1/NF-κB/caspase-9 signaling pathway, suggesting TKL as a promising agent for treating DN.

## Introduction

According to the latest World Health Organization (WHO) report, the number of diabetes mellitus (DM) patients has reached 347 million worldwide, and 114 million of those patients are in China [1]. Diabetic nephropathy (DN) is a serious microvascular complication of DM characterized by initial proteinuria and increased creatinine levels [2]. The incidence of DN is growing at an alarming rate, and DN has now become a worldwide public health problem [3]. The American Diabetes Association reported that 20–40% of patients with type 2 DM will develop DN-related complications ~10 years after their initial diagnosis of DM [4,5]. Among patients who begin receiving renal replacement therapy (RRT), which greatly increases medical costs, the proportion of patients with DN ranges from 24 to 51% [6]. Unfortunately, the current available treatments for DN, such as hypoglycemic drugs, anti-hypertensive agents, and renin–angiotensin system inhibitors, only help to reduce symptoms, and do not significantly delay or halt the progression of DN [7–9]. Therefore, it has become essential to increase our understanding of the pathogenesis of DN and develop new drugs that can delay its progression.

Because the kidney requires high levels of energy for proper function, a state of sustained hyperglycemia with its accompanying glucose-toxicity causes free radicals to accumulate in kidney tubular cells, resulting in increased rates of apoptosis in both the proximal and distal tubular epithelial cells, which is a major feature of diabetic kidney disease [10]. The aspartic acid specific protease caspase-9, encoded by the *CASP9* gene, is an initiator involved in the mitochondrial apoptosis pathway [11]. Hyperglycemia, proteinuria, and angiotensin II all help to activate nuclear factor-κB (NF-κB), which is a ubiquitous transcription factor capable of controlling DNA transcription, cytokine production, and cell survival [12–14]. Evidence suggests that NF-κB is responsible for regulating the expression of genes involved in apoptosis [15]. Blockading the nuclear translocation of NF-κB might attenuate the expression of NF-κB-related genes such as *Bcl-xl* and *Bcl-2*, as well as the cleavage of caspases-9/-3 [16,17]. It has been reported that inhibition of transcription factor RelA (p65), which is an important subunit of the NF-κB complex, contributes to a decrease in retinal ganglion cell apoptosis [18]. Additionally, the phosphorylation of p65 at Ser536 may induce apoptosis in various tumor cells [19]. Herein, we propose that NF-κB p65/caspase-9 signals might be promising therapeutic targets for DN treatment due to their pro-apoptotic effects.

Herbal medicine has been practiced in China for thousands of years, and some herbal medicines have been used for treating DM and its complications, such as DN [20,21]. *Trichosanthes kirilowii* Maxim (TK), also referred to as Tian-Hua Fen, is traditionally used for treating diabetes and its complications in Eastern Asia [22]. Recent pharmacological studies have shown that pre-treatment with a TK extract can attenuate histopathological changes in the kidney and reduce the numbers of apoptotic cells [23]. Evidence indicates that the ability of TK to inhibit tumor growth is likely associated with an inhibition of NF-κB activity [24]. Lectin compounds comprise the main ingredients responsible for the hypoglycemic activity of TK [25]. *Trichosanthes kirilowii* lectin (TKL) is a galactose-specific plant thrombin that not only has the ability to agglutinate blood cells and sperm cells, but also participates in a series of important physiological and pathological processes [26]. In a recent, study, TKL displayed hypoglycemic effects in alloxan-induced diabetic mice [27]; however, no publication has reported the protective effects of TKL against DN.

We used a high-dose glucose (HG)-induced HK2 cell model and a STZ-induced DM rat DN model to investigate how TKL affects the NF-κB p65/caspase-9 signaling pathways. We also discuss the possibility of developing TKL as a novel agent for treating DN.

## Materials and methods

### Chemicals and materials

Cell Counting Kit-8 (CCK-8), Bicinchoninic acid (BCA) Protein Assay Kits, Annexin V-FITC Apoptosis Detection Kits, and Cell Cycle Analysis Kits were all purchased from the Beyotime Institute of Biotechnology (Jiangsu, China). Protein Extraction Kits were obtained from KEYGEN Biotech. Co., Ltd. (Nanjing, China). Diaminobenzidine (DAB) substrate kits were purchased from Zhongshan Golden Bridge Biotechnology (Beijing, China). Terminal deoxynucleotide transferase-mediated dUTP nick-end labeling (TUNEL) kits were provided by Roche Diagnostics (Germany). 4′,6′-Diamidino-2-phenylindole (DAPI), tris (hydroxymethyl) aminomethane (Tris), and sodium dodecyl sulphate (SDS) were purchased from Sigma (St. Louis, MO, U.S.A.). Anti-LOX1, anti-caspase-9, anti-p65, anti-p-IKKβ, anti-IKKβ, anti-p-IkBα, anti-IkBα, anti-GAPDH, anti-β-tubulin, and anti-Lamin B primary antibodies, and horseradish peroxidase-conjugated antibody were obtained from Abcam (Cambridge, U.K.). Plasmids harboring the wild-type caspase-9 response element (WT-luciferase-caspase-9) and the corresponding mutant (MUT-luciferase-caspase-9) were purchased from Vipotion Biotechnology (Guangzhou, China). Pierce Agarose Chip Kits were obtained from Thermo Fisher Scientific (Waltham, MA, U.S.A.).

### Extraction of TKL

TKL was extracted with phosphate-buffered saline (PBS) and purified by dialysis. Briefly, Tian-Hua Fen was added to PBS at a weight to volume ratio of 1:30, and the TKL was extracted in a 4°C refrigerator for 24 h. The mixture was then centrifuged at 4000 rpm for 10 min, and supernatant was collected. Next, the supernatant was added to a 70% ammonium sulfate solution and let sit for 24 h; after which, the lower sediment was harvested by centrifugation at 10,000 rpm. Finally, the ammonium salt in the TKL solution was removed by dialysis, and the TKL extract was dried in a vacuum freeze dryer.

### Cell culture

HK-2 human kidney tubular epithelial cells were purchased from the Institute of Biochemistry and Cell Biology (Shanghai, China) and maintained in low-glucose DMEM supplemented with 10% FBS and antibiotics (100 IU/ml penicillin and 100 mg/ml streptomycin) in a humidified atmosphere of 5% CO_2_.

### TKL toxicity assay

HK-2 cells were cultured in 96-well plates at a density of 1000 cells/well and let grow to 80% confluence; after which, they were treated with different concentrations of TKL for 24 h. CCK-8 solution (10 μl) was added to each well and incubated for another 2 h. The OD value of each well at 450 nm was measured with a microplate reader (Thermo Fisher Scientific, Waltham, MA, U.S.A.).

### High glucose (HG)-induced cell toxicity

Cultured HK-2 cells were divided into the following eight groups based on the cell treatment regimen and cell culture medium: 5 mM glucose (Normal control group); 30 mM glucose (HG group); 30 mM glucose and 62.5 nM TKL (TKL low-dose group); 30 mM glucose and 125 nM TKL (TKL medium-dose group); 30 mM glucose and 250 nM TKL (TKL high-dose group); 30 mM glucose and pre-treated with 250 μg/ml polyinosinic acid (Poly IC); 250 nM TKL (Negative control group); 30 mM glucose and treated with 20 μM gliquidone (GLQ). GLQ has been previously used to treat type 2 DM in the clinic [28]. Thus, the GLQ group was used as a positive control group in these experiments. Poly IC was used as a blocking agent of LOX1, which is the receptor for TKL. GLQ was used as a positive control and has been widely used in the clinical treatment of DN. Cell viability was assessed using the CCK-8 method as described above.

### Apoptosis assay

Cells were collected after being treated with different concentrations of TKL (62.5, 125, or 250 nM), Poly IC, or GLQ for 48 h, and then incubated with Annexin V-FITC and propidium iodide (PI) in binding buffer for 10 min in a dark room according to the manufacturer’s instructions. Cells harvested from each group were analyzed by flow cytometry (Becton-Dickinson, Franklin Lakes, NJ, U.S.A.).

### Cell cycle analysis

HK-2 cells were cultured in six-well plates and treated as described above for the indicated time periods. After treatment, the cells were harvested and re-suspended in a mixture of 25 μl of propidium iodide (50 μg/mL), 10 μl of Rnase A (10 mg/ml), and 500 μl of staining buffer; after which, they were fixed with 70% ethanol for 10 min. The cell cycle profile of HK-2 cells in each group was analyzed with a flow cytometer (Becton-Dickinson, U.S.A.) after incubation at 37°C for 30 min.

### Immunofluorescence assays

Immunofluorescence staining for LOX1, caspase-9, and p65 in HK-2 cells was performed using anti-LOX1, anti-caspase-9, and anti-p65 antibodies, respectively, in a humidified box at 4°C overnight. The immunostained cells were then incubated with a TRITC (red fluorescence) or FITC (green fluorescence)-labeled secondary antibody for 1 h at 37°C. The cell nuclei were then stained with DAPI solution (1.0 μg/ml) for 10 min. Images of immunofluorescence staining were obtained by using a laser confocal microscope (Olympus, Japan).

### Chromatin immunoprecipitation (ChIP) assay

ChIP assays were performed using a Thermo Scientific Pierce Agarose Chip Assay Kit according to the manufacturer’s protocol. Briefly, HK-2 cells were treated with a high concentration of glucose with or without a high dose of TKL. Non-treated cells were used as controls. After 48 h of incubation, the DNA and proteins in cells were cross-linked by addition of 1% formaldehyde to the culture medium. The HK-2 cells were then incubated in glycine solution for 5 min on ice to quench any unreacted formaldehyde. Next, the cells were harvested in 1× PBS solution containing a protease inhibitor cocktail, and then re-suspended in lysis buffer containing protease inhibitors. The cells were then sonicated, and the cellular DNA was analyzed by agarose gel electrophoresis. Dilution buffer containing a protease inhibitor cocktail was added to the sonicated DNA (volume ratio = 9:1) to force immediate immunoprecipitation. One percent of the immunoprecipitated mixture was aliquoted for use as input DNA, while anti-p65 antibody/protein G magnetic bead complexes were added to the remaining solution, which was then incubated for 24 h with rotation at 4°C. Finally, binding of the caspase-9 proximal promoter (CASP-9 PII) to p65 was analyzed using a MiniOptican system (Bio-Rad, Hercules, CA, U.S.A.). The PCR conditions were as follows: pre-incubation at 92°C for 2 min, followed by 40 cycles of denaturation at 95°C for 20 s, annealing at 58°C for 20 s, and extension at 72°C for 20 s. The PCR products were detected by 1.5% agarose gel electrophoresis, with normal human IgG serving as the negative control.

### Dual-luciferase reporter assay

HK-2 cells were cultured in 24-well plates for 24 h and then transfected with a wild-type caspase-9-luciferase reporter plasmid (caspase-9-WT) and pcDNA-p65 by using Lipofectamine 2000 according to the manufacturer’s protocol. HK-2 cells transfected with a mutant caspase-9-luciferase reporter plasmid (caspase-9-MUT), and pcDNA-p65 served as negative control cells. After 6 h of transfection, the cells were incubated with TKL (250 nM) or without TKL for 18 h at 37°C in a 5% CO_2_ incubator. Luciferase activity was assessed using a double-luciferase Reporter Assay Kit (TransGen Biotec, China) and a Dual-Light Chemiluminescent Reporter Gene Assay System (Berthold, Germany) that was specific for Renilla luciferase activity.

### Animals

All experimental procedures conducted with animals were performed in strict accordance with PR of China Legislation Regarding the Use and Care of Laboratory Animals. The animals were allowed to acclimate for 1 week prior to use and were housed in a room with a barrier system under standardized conditions. Male Wistar rats (6–8 weeks, 180–220 g) were injected with a single intraperitoneal (i.p.) dose of STZ (60 mg/kg) in 0.1 M citrate buffer, (pH = 4.5). Control rats were i.p. injected with an equivalent volume of vehicle. At 72 h after STZ administration, the concentrations of fasting tail vein blood glucose, urine volumes, and the urinary protein levels were measured. The rat qualification criteria for inclusion in the STZ-induced DN model required that three parameters reach the following values for three consecutive times: (1) fasting blood glucose level > 16.6 mmol/l, (2) urine volume > 1.5-fold the primary urine volume, and (3) 24-h urinary protein level > 2-fold the primary urinary protein level. Rats included in the DN model were divided into seven treatment groups (*n* = 6 per group): (1) Control group: intragastric administration (i.g.) with normal saline; (2) STZ group: i.p. with STZ and i.g. with normal saline; (3) TKL low-dose group: i.p. with STZ and i.g. with 10 mg/kg/day of TKL; (4) TKL middle-dose group: i.p. with STZ and i.g. with 20 mg/kg/day of TKL; (5) TKL high-dose group: i.p. with STZ and i.g. with 40 mg/kg/day of TKL; (6) negative control group: i.p. with STZ, intramuscular injection with 1 ml/kg/day Poly IC, and i.g. with 40 mg/kg/day of TKL; (7) positive control group: i.p. with STZ and i.g. with 20 mg/kg/day of GLQ. After 8 weeks of administration, the rats were injected into their tail vein with FITC–BSA (10 mg/kg), and then anesthetized with chloral hydrate 30 min later. The left kidney of each rat was stored at −80°C for examination, and the right kidney was fixed in 4% paraformaldehyde after the abdominal aorta had been perfused with PBS.

### Histopathological examination

The kidney was embedded in 4% paraformaldehyde for at least 24 h, and then embedded in paraffin wax and sectioned (4-μm thickness) for hematoxylin–eosin (H&E) and Masson staining. In addition, frozen sections of paraformaldehyde-fixed kidney were also stained with periodic acid–Schiff (PAS). The images were observed under a light microscope (Nikon, Japan) and photographed at 200× magnification.

### TUNEL assay

Paraffin embedded sections of kidney tissue were deparaffinized with xylene and then rehydrated with descending concentrations of alcohol. Apoptotic cells in the kidney tissues were detected by TUNEL staining. In brief, tissue sections were incubated with fluorescein (green)-labeled de-oxyuridine triphosphate solution for 60 min at 37°C, and then stained with DAPI (1 μg/ml). Images of the stained tissue sections were photographed at 400× magnification with a fluorescence microscope (Nikon, Japan).

### Immunohistochemistry assay

Deparaffinized kidney tissue sections were incubated in normal goat serum to block nonspecific protein binding. The sections were then incubated with primary rabbit antibodies including anti-caspase-9, anti-p65, and anti-LOX1 antibodies at a 1:100 dilution, respectively; after which, the sections were incubated with biotin-labeled goat anti-rabbit immunoglobulin G and horseradish peroxidase-conjugated streptavidin for 20 min. Next, the sections were incubated with DAB solution for 20 min and counterstained with hematoxylin. Images were observed and photographed under a light microscope at 400× magnification.

### Quantitative real-time PCR assay

Total RNA was extracted from rat tissues with RNAiso Plus reagent (TaKaRa Biotechnology Co., Ltd., Japan) according to the manufacturer’s instructions. The reverse transcription polymerase chain reaction (RT-PCR) was performed with a PrimeScript^®^ RT reagent Kit (TaKaRa Biotechnology Co., Ltd., Japan) and using a TC-512 PCR system (TECHNE, U.K.). The levels of mRNA expression were quantified by real-time PCR performed with SYBR^®^ PremixEx Taq^™^II (Tli RNaseH Plus) (TaKaRa Biotechnology Co., Ltd., Japan) and an ABI 7500 Real-Time PCR System (Applied Biosystems, Santa Clara, CA, U.S.A.). The sequences of the primers are shown in Supplementary Table S1, and the *GAPDH* gene served as the house-keeping gene.

### Western blot studies

The total, nuclear, and cytoplasmic proteins in HK-2 cells and rat homogenates were extracted according to standard protocols provided with a protein extraction kit (Beyotime, China). Protein concentrations were determined using a BCA protein assay kit. Equal amounts of total protein from each sample were loaded onto SDS-PAGE gels, and the separated protein bands were transferred onto polyvinylidene fluoride (PVDF) membranes (Millipore, Burlington, MA, U.S.A.). The membranes were then blocked with 5% dried skim milk for 3 h at room temperature and incubated overnight at 4°C with primary antibodies including anti-IL-6, anti-IL-18, anti-LOX, anti-caspase-9, anti-p65, anti-p-IKKβ, anti-IKKβ, anti-p-IkBα, anti-IkBα, anti-GAPDH, anti-β-tubulin, and anti-Lamin B, respectively. Next, the membranes were incubated with horseradish peroxidase-conjugated antibody for 2 h at room temperature, and an enhanced chemiluminescence method was used to detect the immunostained protein bands. Images were acquired using a Bio-Spectrum Gel Imaging System (UVP, U.S.A.).

### Statistical analysis

All statistical results are shown as the mean ± standard deviation (SD). Multiple group analyses were performed using one-way analysis of variance (ANOVA) followed by Tukey’s *post-hoc* test. *Post-hoc* tests were performed when the ANOVA indicated that a significant difference existed between groups. Comparisons between two groups were performed using an unpaired Student’s *t*-test. *P*-values < 0.05 were considered statistically significant.

## Results

### TKL inhibited high-glucose (HG)-induced cytotoxicity to HK-2 cells

**Figure 1:**
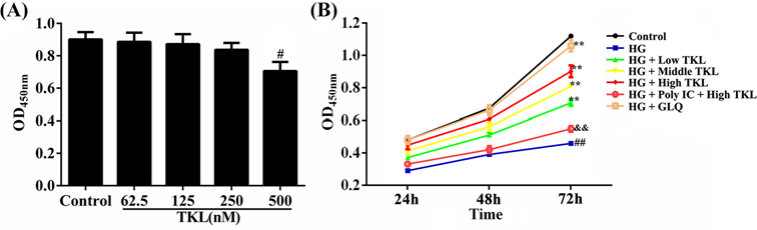
TKL reduced the toxicity of high glucose (HG) to HK-2 cells (**A**) Cell proliferation effect and cytotoxicity of TKL alone on HK-2 cells. HK-2 cells were treated with different doses of TKL, and the cell viability in each group was evaluated with a Cell Counting Kit-8 kit (*n*=6, ^#^*P*<0.05 vs. control group). (**B**) TKL dose-dependently increased the viability of HK-2 cells treated with HG (*n*=6, ^##^*P*<0.01 vs. control group; ^**^P<0.01 vs. HG group; ^&&^*P*<0.01 vs. high-dose TKL group). GLQ was used as a positive control. Data are presented as the mean ± SD.

The effect of TKL on the viability of HK-2 cells was tested using the CCK-8 assay (Figure 1). When compared with the control group, there were no significant changes in the viability of HK-2 cells treated with TLK at concentrations of 62.5–250 nM for 24 h, as indicated by the OD_450_ values. However, treatment with 500 nM TKL significantly decreased the viability of HK-2 cells. These results indicated that HK-2 cells were not adversely affected by a TKL concentration <250 nM (Figure 1A). Therefore, we chose 62.5, 125, and 250 nM as the low, medium, and high doses of TKL, respectively, for use in subsequent experiments. We then tested the effects of TKL on HG-treated HK-2 cells (Figure 1B) and found that their viability was decreased by treatment with 30 mM HG for 24, 48, or 72 h. However, TKL (62.5, 125, and 250 nM) and GLQ (20 μM) notably increased those OD_450_ values when compared with OD_450_ values in the HG group. The viability of cells pre-treated with Poly IC (250 μg/ml) and 250 nM TKL was much lower than that of cells in the high-dose TKL group, indicating that TKL could protect HK-2 cells against HG-induced down-regulation of their viability.

### TKL dose-dependently inhibited cell apoptosis in HG-treated HK-2 cells

**Figure 2:**
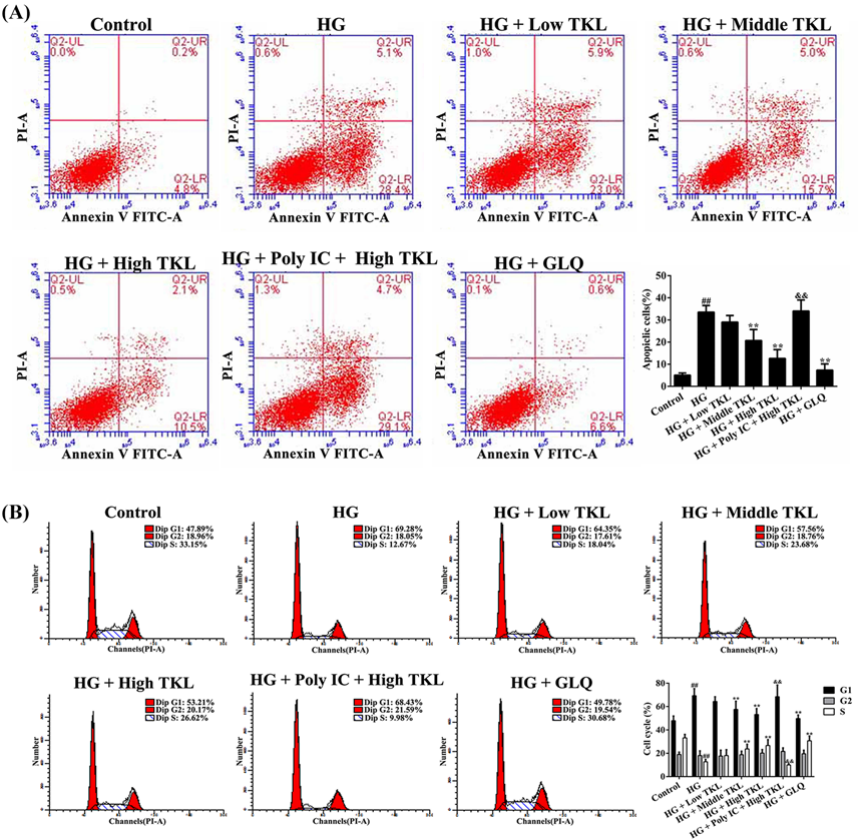
TKL inhibited high glucose (HG)-induced apoptosis and decreased DNA synthesis in HK-2 cells (**A**) TKL inhibited the apoptosis of HG-treated HK-2 cells in a dose-dependent manner. The apoptotic cells were analyzed by flow cytometry. (**B**) TKL inhibited the decrease in DNA synthesis in HG-treated HK-2 cells. The profile of DNA content was obtained by cytometry. The shift from G1 to S phase among HG-treated cells could be reversed by TKL. Data are presented as the mean ± SD; *n*=3, ^##^*P*<0.01 vs. control group; **P<0.01 vs. HG group; ^&&^*P*<0.01 vs. high-dose TKL group.

To investigate the effects of HG on HK-2 cell apoptosis as well as the effects of TKL intervention, the apoptosis rates of HK-2 cells in different groups were analyzed by flow cytometry. The results showed that HG treatment significantly increased the apoptotic rate of HK-2 cells (33.5%) when compared with that of control cells (5%) (Figure 2A). However, the apoptotic rates were notably decreased to 28.9%, 20.7%, and 12.6% among HK-2 cells treated with TKL concentrations of 62.5, 125, and 250 nM, respectively. The apoptotic rate of GLQ-treated cells was notably decreased to 7.2%. Moreover, the number of apoptotic cells was markedly increased after pre-treatment with Poly IC (250 μg/ml) and 250 nM TKL when compared with the number of apoptotic cells in the high-dose TKL group. These results indicated that TKL could protect HG-treated HK-2 cells by inhibiting their apoptosis.

### TKL could counteract HG-induced G1 arrest

An analysis of cell cycling by flow cytometry (Figure 2B) showed that HG-treated HK-2 cells were arrested at the G1 phase; however, this scenario could be reversed by treatment with TKL (62.5, 125, or 250 nM) for 48 h, indicating that TKL could counteract HG-induced G1 phase arrest. However, this effect of TKL vanished in HK-2 cells pre-treated with Poly IC, which is an inhibitor of the membrane protein LOX1. GLQ was used as a positive control that also strongly inhibits HG-induced G1 arrest. These results showed that the effect of TKL against HG-induced HK-2 cell cycle arrest was mediated by LOX1.

### Poly IC inhibited LOX1 expression in HG- and TKL-treated HK-2 cells

**Figure 3:**
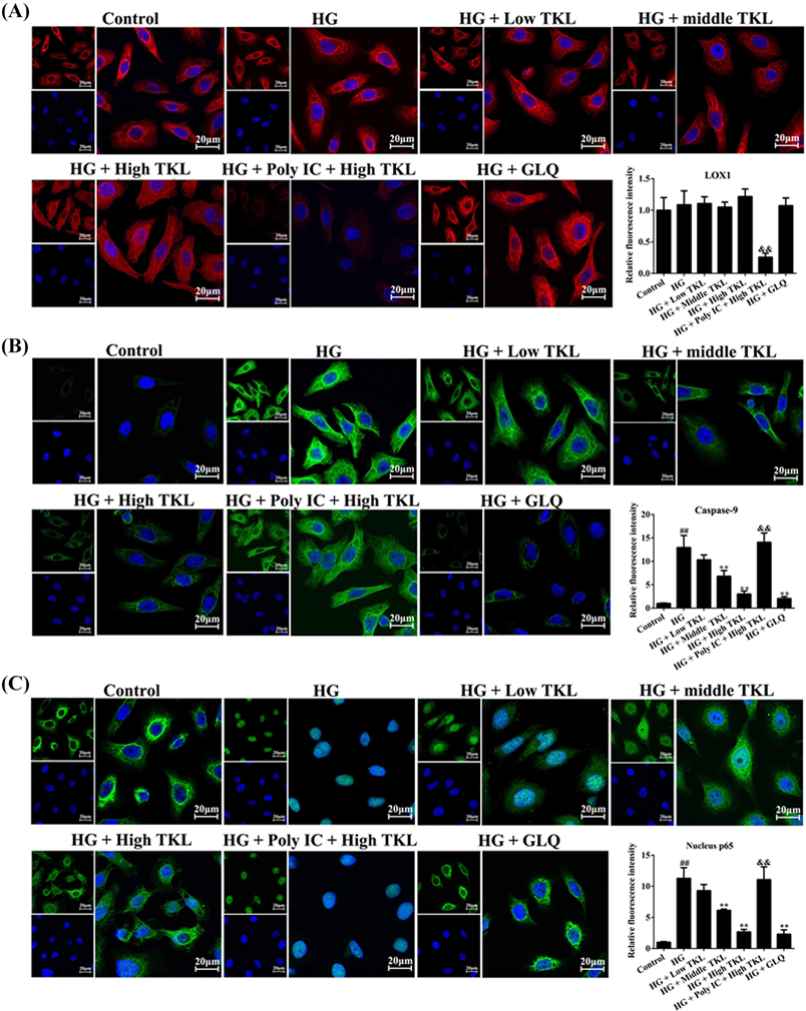
TKL inhibited the high glucose (HG)-induced overexpression of cytosolic caspase-9 and nuclear translocation of p65 via endocytosis pathways in HK-2 cells (**A**) Poly IC strongly inhibited LOX1 protein expression, whereas HG, TKL, GLQ, and their indicated combinations had no obvious effects. The fluorescence intensity (red) indicated the expression level of LOX1 in HK-2 cells. Red and blue fluorescence represent LOX1 and the cell nucleus, respectively. (**B**) TKL inhibited HG-induced up-regulation of caspase-9 protein expression through LOX1-mediated endocytosis pathways. HK-2 cells were treated with different concentrations of TKL and/or HG, and expression of caspase-9 (green) was visualized under a confocal microscope. Fluorescence intensity (green) indicate the level of caspase-9 expression. Blue fluorescence indicates the cell nucleus. (**C**) TKL inhibited the nuclear translocation of p65 in HG-treated HK-2 cells. GLQ was used as a positive control. Green and blue fluorescence represent p65 and the cell nucleus, respectively. Data are presented as the mean ± SD; *n*=3, ^##^*P*<0.01 vs. control group; **P<0.01 vs. HG group; ^&&^*P*<0.01 vs. high-dose TKL group.

Poly IC is a potent inhibitor of the membrane protein LOX1, which is a receptor that contains a TKL-like binding domain at its C-terminus. In order to study whether TKL exerts its protective effect against DN via its receptor LOX1 or its related cell signaling pathways, we used Poly IC as an inhibitor to reduce LOX1 expression. Confocal microscopy data showed that when compared with the control group, no significant changes in LOX1 expression could be seen in the groups treated with HG, HG plus different concentrations of TKL, or HG plus GLQ. However, pre-treatment with Poly IC dramatically reduced the levels of LOX1 expression (red) (Figure 3A), indicating Poly IC could inhibit LOX1 expression in HG- and TKL-treated HK-2 cells.

### The TKL-induced down-regulation of caspase-9 expression in HG-treated HK-2 cells involved the LOX1/caspase-9 pathways

Caspase-9 is a key molecule involved in apoptosis [29]. To understand whether TKL’s ability to protect against the toxic effects of high-glucose treatment was related to caspase-9, we performed immunofluoresence assays to examine the relative levels of caspase-9 expression in HG-treated HK-2 cells with or without different concentrations of TKL (Figure 3B). Confocal microscopy data showed that HG (30 mM) markedly increased caspase-9 (green) expression in HK-2 cells, whereas supplemental TKL (62.5, 125, or 250 nM) or GLQ (20 μM) did not significantly inhibit HG-induced caspase-9 overexpression. However, the inhibitory effects of TKL on HG-induced caspase-9 expression in HK-2 cells could be blocked by Poly IC, as shown by the finding that caspase-9 expression was significantly increased in high-dose TKL treated HG-treated cells that were pre-treated with Poly IC (20 μM).

### TKL-induced nuclear translocation of p65 in HG-treated HK-2 cells involved the LOX1/caspase-9 signaling pathway

In normal cells, p65 expression is mainly found in the cytosol; however, p65 can translocate into the nucleus and regulate gene transcription by targeting gene promoter regions. To understand whether and how TKL influences p65 translocation, we detected the distribution of p65 in HG-treated cells by confocal microscopy. As shown in Figure 3C, HG markedly induced p65 protein translocation from the cytoplasm into the nucleus (green), and that translocation could be inhibited by TKL and GLQ. However, pre-treatment with Poly IC, an inhibitor of LOX1, halted the nuclear translocation of p65 in high-dose TKL and HG-treated HK-2 cells. Taken together, our data showed that TKL facilitated a potent inhibition of p65 nuclear translocation via LOX1-mediated signaling pathways.

### TKL inhibited the binding of p65 to CASP9 in HG-treated HK-2 cells

**Figure 4:**
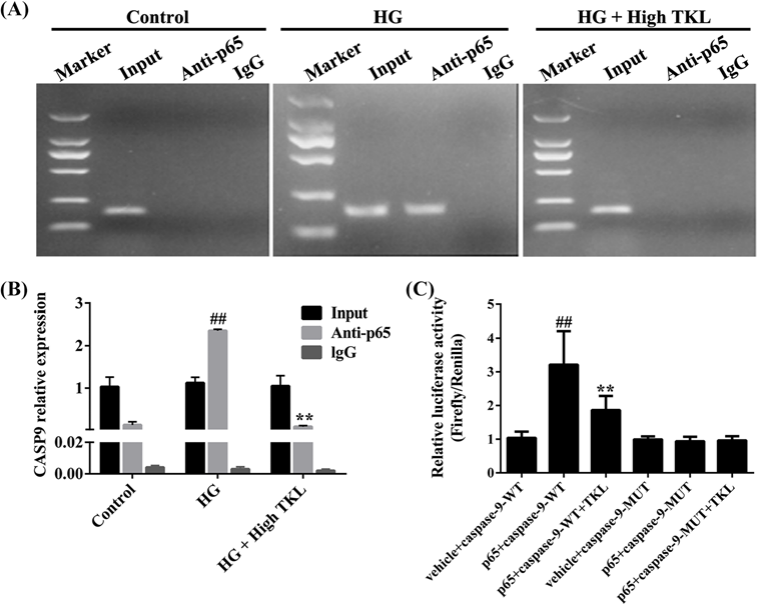
TKL inhibited the binding of p65 to the *CASP9* gene (caspase-9 protein coding) in HG-treated HK-2 cells (**A**) TKL specifically inhibited HG-induced binding of p65 to the *CASP9* gene in HK-2 cells. The bound *CASP9* gene was analyzed by agarose gel electrophoresis. IgG was used as a negative control for the p65 antibody. (**B**) TKL inhibited HG-induced *CASP9* expression in HG-treated HK-2 cells after pull-down p65. *CASP9* gene expression was quantified by real-time PCR (*n*=3, ^##^*P*<0.01 vs. control group; **P<0.01 vs. HG group). (**C**) TKL inhibited *CASP9* gene expression in HG-treated HK-2 cells, as shown by decreased relative luciferase activity in the p65 + caspase-9-WT + TKL group. Caspase-9-MUT was used as a negative control (*n*=3, ^##^*P*<0.01 vs. ‘vehicle + caspase-9-WT’ group; **P<0.01 vs. ‘p65 + caspase-9-WT’ group). Data are presented as the mean ± SD.

As described above, we found that TKL could inhibit HG-induced caspase-9 overexpression and also p65 translocation from the cytosol into the cell nucleus. We then performed ChIP assays to determine whether TKL could inhibit the binding of p65 to *CASP9* (caspase-9 coding gene) in HG-treated HK-2 cells. Our results showed that in HG-treated HK-2 cells, p65 binds to CASP9, as shown by the fact that the *CASP9* gene could be pulled down by anti-p65 antibody. However, the p65 that bound to the *CASP9* gene in response to HG treatment became disassociated in the presence of TKL (250 nM), as no *CASP9* gene band could be detected in TKL- and HG-treated cells (Figure 4A). Real-time PCR results also showed that HG-induced CASP9 expression, which was decreased by TKL (Figure 4B). These results indicated that TKL inhibited the binding of p65 protein to the *CASP9* gene.

### TKL inhibited *CASP9* gene transcription in HG-treated HK-2 cells

Since TKL inhibited the binding of p65 to *CASP9*, we speculated that TKL might inhibit *CASP9* gene transcription in HG-treated HK-2 cells. To test this hypothesis, we transfected HK-2 cells with either p65/caspase-WT or p65/caspase-MUT in the presence or absence of TKL (Figure 4B). The luciferase activity of HK-2 cells was significantly increased by p65 overexpression and transfection with the wild-type caspase-9 constructs (caspase-9-WT), indicating that the binding of p65 had enhanced *CASP9* transcription. However, TKL significantly decreased the luciferase activity of cells co-transfected with p65 and caspase-9-WT. This suggests that TKL induced the disassociation of p65 from *CAPS9*, and thereby inhibited transcription of the *CASP9* gene, resulting in a down-regulation of luciferase activity. This result was further verified by the use of caspase-9-MUT. In summary, TKL inhibited the transcription of *CASP9* in HG-treated HK-2 cells.

### The protective effects of TKL on HG-treated HK-2 cells was mediated by the LOX1/IKKβ/IkBα/p65/caspase-9 signaling pathways

**Figure 5:**
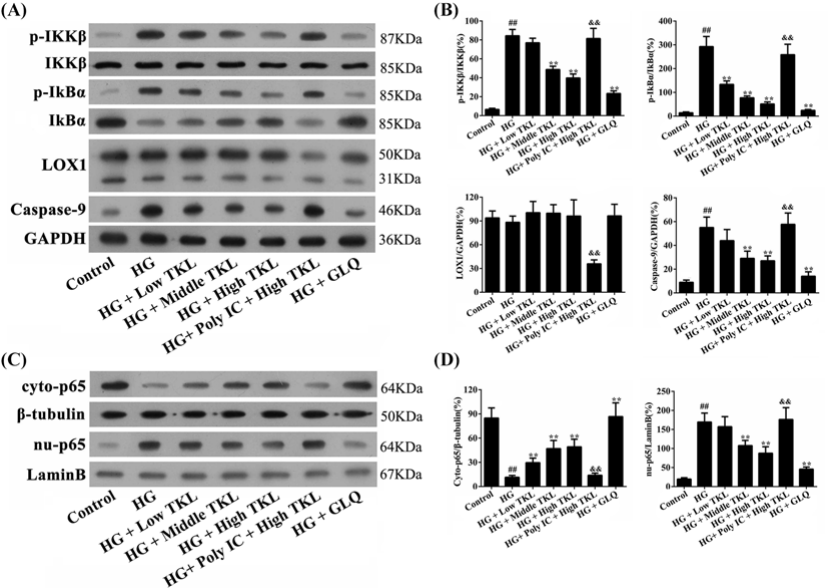
Expression profile of the LOX1/IKK/caspase-9/p65 signaling pathway in HK-2 cells (**A**) and (**B**) Effect of TKL on the expression of IKKβ, IkBα, LOX1, and caspase-9 proteins in HK-2 cells. The relative expression levels of the indicated proteins in cell homogenates were analyzed by Western blot assays, and the results were normalized to those for GADPH. (**C**) and (**D**) Effects of TKL on p65 nuclear translocation in HG-treated HK-2 cells. It is noteworthy that the decrease in cytosolic p65 (cyto-p65) corresponded to the increase in nuclear p65 (nu-p65). TKL significantly inhibited HG-induced p65 nuclear translocation, whereas Poly IC, a LOX1 inhibitor, reduced the inhibition caused by TKL. Data are presented as the mean ± SD; *n*=3, ^##^*P*<0.01 vs. control group; **P<0.01 vs. HG group; ^&&^*P*<0.01 vs. high-dose TKL group.

It has been reported that the p65 signaling pathway involved in activation of IKKβ and IκBα plays key roles in facilitating HG-induced damage to HK-2 cells [30]. We investigated the expression profiles of key components in the p65/caspase-9-mediated signaling pathway in HG-treated HK-2 cells and also the interventional effects of TKL by Western blotting. Our data showed that HG significantly up-regulated the expression levels of active IKKβ (p-IKKβ), active IκBα (p-IκBα), and caspase-9, all of which could be down-regulated by TKL and GLQ (Figure 5A,B). However, the effects produced by HG-induced up-regulation of p-IKKβ, p-IκBα, and caspase-9, and down-regulation of IκBα expression in HK-2 cells could be attenuated by treatment with a high dose of TKL, whereas the inhibitory effects of TKL could be counteracted by pre-treatment with Poly IC. Among those treatments, only Poly IC, a specific inhibitor of the membrane protein LOX1, could significantly inhibit LOX1 protein expression in HG-treated cells. In addition, we found that HG markedly increased the translocation of p65 from the cell cytosol to the nucleus, as shown by a down-regulation of p65 levels in the cytoplasm (cyto-p65), and a corresponding up-regulation of p65 levels in the nucleus (nu-p65) (Figure 5C,D). Moreover, TKL and GLQ could markedly inhibit HG-induced p65 nuclear translocation. However, pre-treatment of HK-2 cells with Poly IC significantly attenuated the protective effect of high-dose TKL against HG treatment. These results demonstrated that TKL exerts its potent inhibitory effects via the LOX1-mediated IKKβ/IkBα/p65/caspase-9 cell signaling pathways.

### TKL reduced histopathological changes in renal tissue in the STZ-induced nephropathy rat model

**Figure 6:**
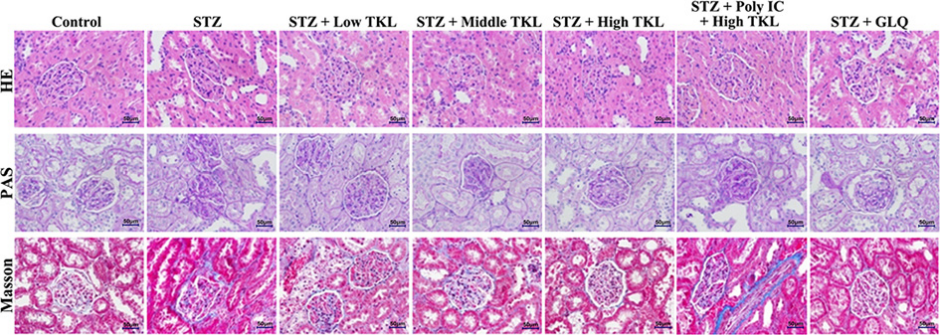
TKL ameliorated renal pathological injury in STZ rats TKL reduced the histopathological changes in renal tissue seen in the STZ-induced nephropathy model, as shown by H&E, PAS, and Masson staining (400× magnification).

We detected the blood glucose levels in rats, and the results are shown in Supplementary Figure S1. When compared with glucose levels in the control group, the blood glucose levels in all of the other groups were significantly increased, indicating that a rat model of STZ-induced nephropathy had been successfully established (Supplementary Figure S1A). However, after the pre-treatments, we found that rats in the TKL and GLQ groups could markedly decrease their blood glucose levels. Moreover, when compared with glucose levels in the high-dose TKL group, the blood glucose levels increased after pre-treatment with Poly IC (Supplementary Figure S1B). In order to determine whether TKL could help protect against DN *in vivo*, histological changes in rat kidney tissues were examined under a microscope after H&E, PAS, and Masson staining (Figure 6). H&E staining showed that STZ could induce dramatic histopathology changes, including partial renal tubular epithelial vacuole degeneration or hyaline degeneration, and the infiltration of inflammatory cells. PAS staining showed that the glomerular basement membranes in kidneys of rats in the STZ-treated group were thicker than those of rats in the control group, and that extracellular matrix had accumulated in the mesangial region. Additionally, when compared with the normal control rats, there was a significant increase in inflammatory cell infiltrates in the interstitium and collagenous fibers of rats in the STZ group. The above histopathology changes could be reversed by TKL and GLQ. Furthermore, there were remarkable histological alterations after pre-treatment with Poly IC when compared with histopathological characteristics in the high-dose TKL group. Taken together, these results indicated that TKL could alleviate DN *in vivo*.

### TKL inhibited cell apoptosis in the kidney tissues of STZ-treated rats

**Figure 7:**
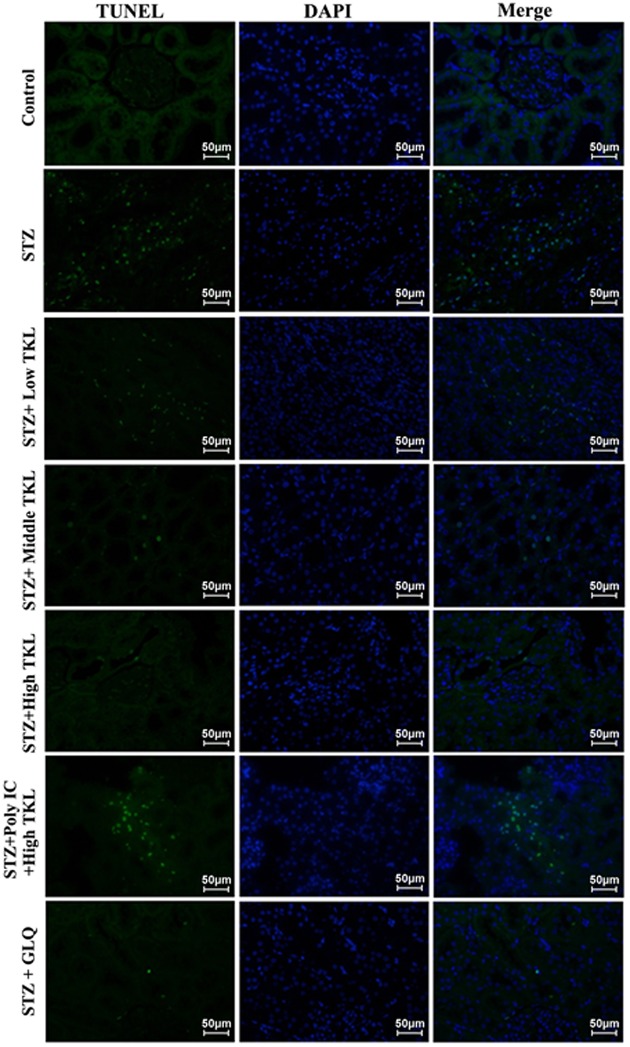
TKL reduced the numbers of apoptotic cells in samples of renal tissue from the STZ-induced nephropathy model, as shown by TUNEL staining (400× magnification) The green and blue fluorescence represent the apoptosis-positive cells and cell nucleus, respectively.

In order to understand whether TKL protects STZ-treated rats by affecting cell apoptosis, TUNEL staining was conducted to identify apoptotic cells in injured kidney tissues. More TUNEL-positive cells with green fluorescence were observed in the STZ-treated group than in the control group. However, TKL and GLQ markedly decreased the numbers of TUNEL-positive cells when compared with those numbers in the STZ group (Figure 7). Moreover, when compared with the high-dose TKL group, the number of TUNEL-positive cells increased after pre-treatment with Poly IC. These data showed that TKL inhibited cell apoptosis in the kidney tissues of STZ-treated rats.

### TKL inhibited caspase-9 expression, the nuclear translocation of p65, and expression of inflammatory markers in STZ-treated rats via LOX1/p65/caspase-9 signaling

**Figure 8:**
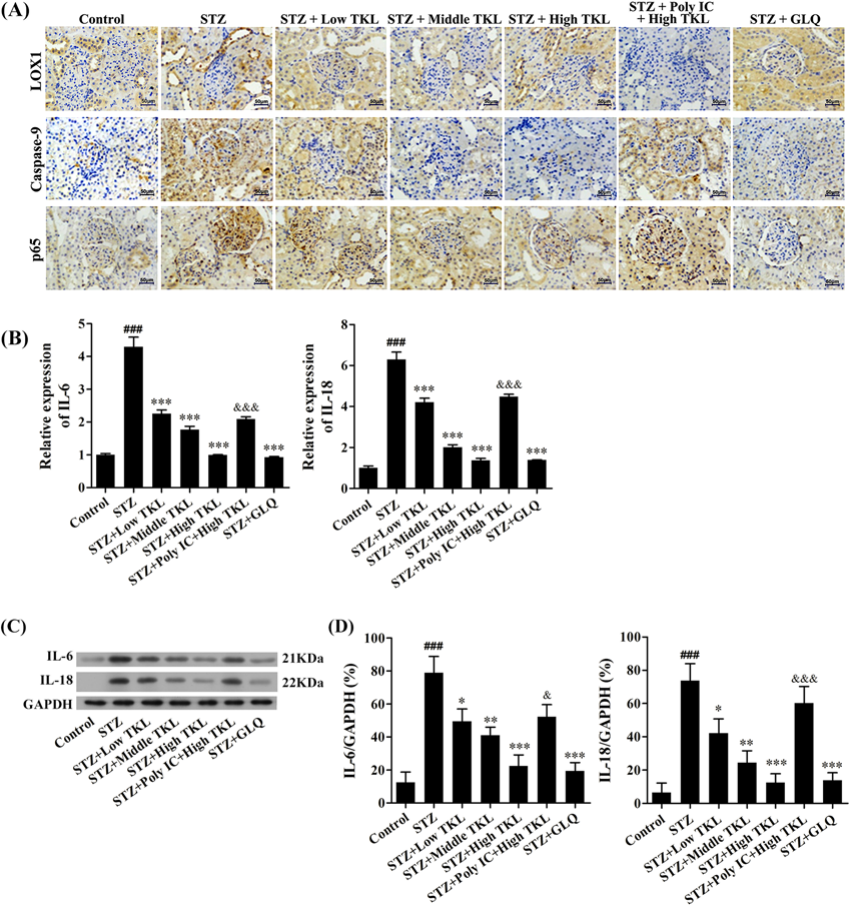
TKL inhibited caspase-9 expression, the nuclear translocation of p65, and the levels of inflammatory markers in STZ-treated rats via LOX1/p65/caspase-9 signaling (**A**) Morphological evidence showing that TKL inhibited caspase-9 expression and the nuclear translocation of p65 in STZ-treated rats via LOX1/p65/caspase-9 signaling. Poly IC is an inhibitor of the LOX1 receptor, which mediates endocytosis. GLQ served as a positive control. The brown and blue areas represent areas of positive protein expression and the cell nucleus, respectively. (**B**) Effect of TKL on mRNA expression for IL-6 and IL-18 in STZ-treated rat kidneys. (**C**) Effect of TKL on the expression of IL-6 and IL-18 proteins in STZ-treated rat kidneys. (**D**) Densitometric analysis of IL-6 and IL-18 protein expression normalized to GAPDH content. Data are presented as the mean ± SD; *n*=3, *P<0.05, **P<0.01 vs. STZ group; ###*P*<0.001 vs. control group; &&&*P*<0.001 vs. high-dose TKL group; ***P<0.001 vs. STZ group; &*P*<0.05 vs. high-dose TKL group.

To further understand the molecular mechanism underlying TKL’s ability to protect against high glucose-induced DN, we conducted an *in vivo* study to detect the expression levels of LOX1, caspase-9, and p65 proteins in the STZ-induced DN model (Figure 8A). The immunohistochemistry data showed that pre-treatment with Poly IC dramatically inhibited LOX1 expression (brown) in the STZ-treated rats. The effects of TKL on caspase-9 expression and the nuclear translocation of p65 in STZ-treated rats were also detected by immunohistochemistry. The results showed that the intensity of caspase-9 staining (brown), which indicates the expression level of caspase-9, observed in the STZ group was much stronger than that in the control group, and STZ-induced caspase-9 overexpression in the rat DN model could be inhibited by TKL or GLQ treatment. However, the inhibitory effect of TKL on STZ-induced caspase-9 expression could be blocked by pre-treatment with Poly IC (Figure 8A). In addition, when compared with the control group, STZ markedly induced p65 protein nuclear translocation from the cytoplasm into the nucleus, and this translocation was significantly decreased by TKL or GLQ. Interestingly, the inhibitory effect of TKL on p65 nuclear translocation vanished after pre-treatment with Poly IC. Taken together, this morphological evidence indicates that TKL’s ability to inhibit STZ-induced caspase-9 overexpression and p65 nuclear translocation is mediated by LOX1/p65/caspase-9 signaling. In order to further detect the renoprotective effect of TKL, we determined the IL-6 and IL-18 mRNA and protein levels in kidney tissues. As shown in Figure 8B,C, STZ markedly up-regulated the levels of IL-6 and IL-18 mRNA and protein, whereas both of those levels were significantly down-regulated in the TKL and GLQ groups. Furthermore, the anti-inflammatory effect of TKL vanished after pre-treatment with Poly IC.

### TKL alleviated diabetic nephropathy (DN) in STZ rats by inhibiting the NF-κB/caspase-9 signaling pathway

**Figure 9:**
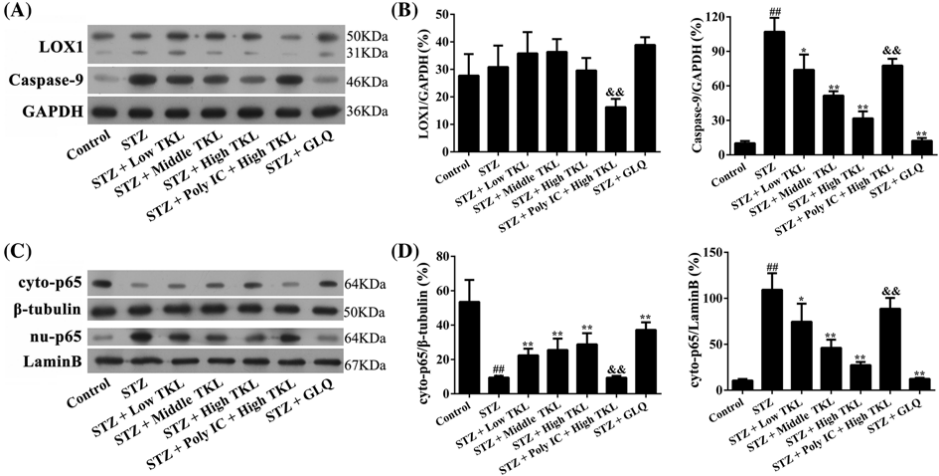
TKL inhibited the NF-κB/caspase-9 signaling pathway in STZ-treated rats (**A**) and (**B**) Effect of TKL on LOX1 and caspase-9 protein expression in STZ-treated rat kidneys. (**C**) and (**D**) Effect of TKL on the nuclear translocation of p65 in STZ-treated rat kidneys. Data are presented as the mean ± SD; *n*=3, ^##^*P*<0.01 vs. control group; **P<0.01 vs. STZ group; ^&&^*P*<0.01 vs. high-dose TKL group; *P<0.05 vs. STZ group.

To further determine the effects of TKL on NF-κB/caspase-9 signaling in our STZ-induced rat DN models, we examined the relative levels of LOX1, caspase-9, cytosol p65, and nuclear p65 expression in STZ-treated rats by Western blotting. The results showed that LOX1 expression levels remained unchanged in the groups pre-treated with TKL or GLQ, but not in the group pre-treated with Poly IC (Figure 9A,B). Meanwhile, Western blotting data also showed that caspase-9 expression was dramatically up-regulated by STZ treatment and could be down-regulated by intervention with TKL or GLQ. Furthermore, the inhibitory effect of high-dose TKL on STZ-induced caspase-9 overexpression could be blocked by pre-treatment with Poly IC, which is potent inhibitor of LOX1. We also found that STZ markedly reduced the levels of cytosolic p65 (cyto-p65), and increased p65 levels in the nucleus (nu-p65), which showed that SZT treatment could induce p65 protein translocation from the cytoplasm into the nucleus. TKL and GLQ could significantly inhibit STZ-induced nuclear translocation. However, the inhibitory effect of TKL on STZ-induced p65 nuclear translocation in the rat DN models could be blocked by pre-treatment with Poly IC (Figure 9C,D). Therefore, TKL also inhibited the NF-κB/caspase-9-mediated signaling pathway in STZ-treated rats.

## Discussion

 DN is the leading cause of end-stage renal failure [2] and frequently occurs due to its risk factors such as hyperglycemia, hypertension, dyslipidemia, and smoking; however, the exact pathogenesis of DN remains unclear [1]. Epidemiological studies have shown that combined end-stage renal failure and diabetes increase the risk for cardiovascular events and premature death [3]. Therefore, there is an urgent need to develop new and effective strategies for treating DN.

In the present study, we used HK-2 cells to examine the effects of TKL on HG-induced cell damage, which is a commonly used *in vitro* model of DN [31,32]. CCK-8 assay results showed that exposure of HK-2 cells to 30 mM glucose produced a significant decrease in cell viability. However, TKL could dose-dependently inhibit the decrease in viability produced by a high glucose concentration. Cell apoptosis and cell cycle data showed that TKL could remarkably inhibit HG-induced apoptosis and cell cycle arrest at the G1 phase. *In vivo* experiments demonstrated that TKL could reduce STZ-induced cell damage and inhibit STZ-induced cell apoptosis. IL-6 and IL-18 are pro-inflammatory cytokines generated by proximal renal tubular cells and have been proven to play important roles in DN [33]. Our study showed that STZ markedly up-regulated the levels of IL-6 and IL-18 mRNA and protein, which were significantly down-regulated by TKL. Thus, we conclude that TKL can alleviate DN *in vitro* and *in vivo* by inhibiting cell apoptosis, and the protective effects of high dose (250 nM) TKL against apoptosis are as strong as those of GLQ.

Previous evidence showed that NF-κB, a pivotal transcription factor, is a heterodimer consisting of p50 and RelA/p65 subunits. Dysfunction of NF-κB is involved in various diseases [34]. In nonstimulated cells, NF-κB p65 locates in the cytoplasm where it is bound to its inhibitor protein IκBα; however, the p65 subunit dissociates from the NF-κB p65 complex and translocates to the nucleus in response to cell insults produced by a high glucose concentration [35]. The phosphorylation and proteolysis of IκBα were mainly due to the increased levels of phosphorylated IκB kinase β (IKKβ), which is the most important kinase upstream of NF-κB p65 [36,37]. In line with previous studies, our data demonstrated that TKL can strongly inhibit the phosphorylation of IKKβ and IκBα, and subsequently reduce the nuclear translocation of NF-κB p65 (p65). In addition, p65 might also regulate the expression of several genes involved in apoptosis, mainly through its tight binding to the *CASP9* gene, and subsequent influence on caspase-9 expression [37,38]. We propose that p65/caspase-9 signals may be attractive targets for treating DN, due to their pro-apoptotic activity. This study demonstrated that the expression levels of caspase-9 were dramatically increased in HG-treated HK-2 cells and the STZ-treated DN rat model, whereas pre-treatment with TKL could reverse those changes. ChIP assay data showed that TKL markedly inhibited the HG-induced binding of p65 to *CASP9*, and the dual-luciferase reporter assay proved that TKL could significantly reduce *CASP9* gene transcription in HG-treated HK-2 cells. These results suggest that TKL can decrease the binding of molecules to *CASP9* DNA. This decreased binding is followed by a down-regulation of caspase-9 protein expression in HG-treated HK-2 cells or STZ-treated rat DN models.

Previous investigators proved that LOX1 is involved in LDL internalization [1,39]. Although our results did not show an increased expression of LOX1 in HG-treated cells, we found that LOX1 expression in HG-treated cells exposed to a high dose of TKL could be significantly down-regulated by pre-treatment with Poly IC, which is a specific inhibitor of LOX1, which contains a binding domain for TKL. After treatment with Poly IC at a concentration that inhibits LOX1, the downstream effects of TKL on HG-treated cells or STZ-treated rat models, such as decreases in p-IKKβ, p-IkBα, and caspase-9 expression, and p65 nuclear translocation, as well as increased levels of un-phosphorylated IkBα, could reversed by inhibition of LOX1 expression with Poly IC. Furthermore, the effects of TKL on the inhibition of p-IKKβ, p-IkBα, and caspase-9 expression, and p65 nuclear translocation were dose dependent. Therefore, we can conclude that the protective effects of TKL against HG- or STZ-induced toxicity were mediated by LOX1-related endocytosis activity, and probably not by changes in LOX1 expression. However, we cannot exclude the possibility that endocytosis of TKL into the cells might mediate LOX1 internalization.

GLQ has been used to treat type 2 DM in the clinic [28]. Our results indicate that TKL and GLQ behave in similar manners when used to treat DM.

**Figure 10:**
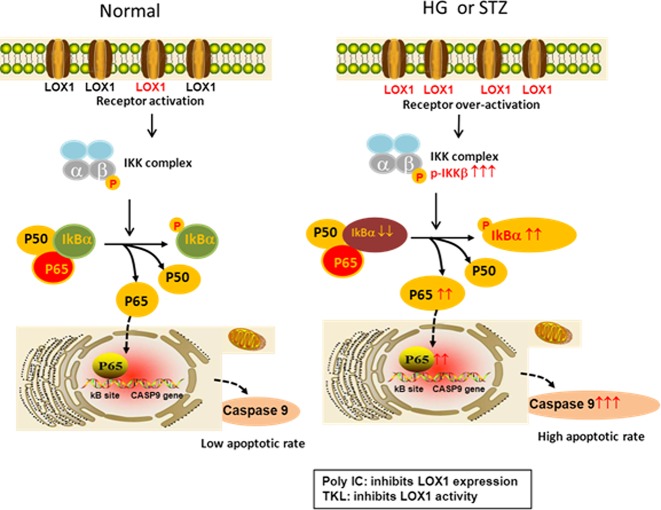
Diagram showing the mechanism by which TKL alleviates DN by inhibiting the NF-κB p65/caspase-9 signaling pathway Under normal conditions, the membrane protein LOX1 functions as a receptor involved in endocytosis, and its activity is low. Three subunits (p65, p50, and phosphorylated IkBα) bind together to form a NF-κB complex. Phosphorylation of the p-IKK complex, which is induced by LOX1 receptor activation, causes phosphorylation of IkBα, resulting in disassociation of p65 from the IkBα complex. The released p65 is then transported into the nucleus, where it binds to the kB site in the promoter region of the *NF-κB* gene to regulate its expression and enhance expression of the *CASP9* gene (Supplementary Figure S1). The phosphorylation of p-IKKβ and p-IkBα, the nuclear translocation of p65, and expression of caspase-9 remain at basal levels. However, high glucose (HG) or STZ-treatment might induce the overactivation of LOX1, and thereby dramatically increase the overexpression of phosphorylated p-IKKβ and p-IkBα. This leads to an increase in free p65 and its robust nuclear translocation, as well as its increased binding to the *CASP9* gene and resultant overexpression of caspase-9 protein. Consequently, both HG- and STZ-treatment can induce high apoptotic rates *in vitro* and *in vivo*. Because there is a binding site for TKL on LOX1, and the inhibitory effects of TKL on p-IKKβ and p-IkBα expression, p65 nuclear translocation, and the subsequent expression of caspase-9 are dose dependent, all those effects are independent of LOX1 expression. In contrast, a down-regulation of LOX1 expression induced by Poly IC can significantly counteract TKL’s protective effects against HG or STZ. These results indicate that TKL most likely exerts its protective effect by regulating LOX1 activity, although the possibility of LOX1-mediated TKL endocytosis cannot be ruled out.

In summary, TKL protected against DN progression by inhibiting the LOX1/NF-κB p65/caspase-9 signaling pathway (Figure 10). It is worthwhile to consider TKL as a potential new agent for treating DN. Nevertheless, further studies are required to understand the detailed mechanism underlying the function and clinical applications of TKL.

### Supporting information

**Figure S1.** TKL markedly decreased the blood glucose levels in STZ-induced nephropathy rat models. (A) The blood glucose levels in rats of different groups before the treatments; (B) The blood glucose levels in rats of different groups after the treatments. Data are presented as mean ± SD; n=6, ^##^p<0.01 versus control group; ^*^p<0.05 versus STZ group; ^&^p<0.05 versus high-dose TKL group.

**Table S1:** The primer sequences used for real-time PCR assay in rats.

## Abbreviations

BCA: bicinchoninic acid
CCK-8: cell counting kit-8
CHIP: chromatin immunoprecipitation
DAB: diaminobenzidine
DAPI: 4′,6′-diamidino-2-phenylindole
DM: diabetes mellitus
DN: diabetic nephropathy
GLQ: gliquidone
H&E: hematoxylin–eosin
HG: high glucose
IKKβ: IκB kinase β
LOX1: lectin-like oxLDL receptor 1
NF-κB: nuclear factor-kappa B
PAS: periodic acid-schiff
Poly IC: polyinosinic acid
RRT: renal replacement therapy
SDS: sodium dodecyl sulphate
STZ: streptozocin
TKL: *Trichosanthes kirilowii* lectin
TUNEL: terminal deoxynucleotide transferase-mediated dUTP nick-end labeling

## References

[1] Bhutani J. and Bhutani S. (2014) Worldwide burden of diabetes. Indian J. Endocrinol. Metab. 18, 868–870 10.4103/2230-8210.141388 25364686 PMC4192997

[2] Chopra B. and Sureshkumar K.K. (2014) Co-stimulatory blockade with belatacept in kidney transplantation. Expert Opin. Biol. Ther. 14, 563–567 10.1517/14712598.2014.896332 24620724

[3] Xue R., Gui D., Zheng L., Zhai R., Wang F. and Wang N. (2017) Mechanistic insight and management of diabetic nephropathy: recent progress and future perspective. J. Diabetes Res. 2017, 1839809 10.1155/2017/1839809 28386567 PMC5366800

[4] Saran R., Li Y., Robinson B., Ayanian J., Balkrishnan R., Bragg-Gresham J. (2015) US Renal Data System 2014 Annual Data Report: Epidemiology of Kidney Disease in the United States. Am. J. Kidney Dis. 66, S1–S305 10.1053/j.ajkd.2015.05.001 PMC6643986 26111994

[5] de Boer I.H., Rue T.C., Hall Y.N., Heagerty P.J., Weiss N.S. and Himmelfarb J. (2011) Temporal trends in the prevalence of diabetic kidney disease in the United States. JAMA 305, 2532–2539 10.1001/jama.2011.861 21693741 PMC3731378

[6] van Dijk P.R., Kramer A., Logtenberg S.J., Hoitsma A.J., Kleefstra N., Jager K.J. (2015) Incidence of renal replacement therapy for diabetic nephropathy in the Netherlands: Dutch diabetes estimates (DUDE)-3. BMJ Open 5, e5624 10.1136/bmjopen-2014-005624 PMC4316478 25636789

[7] Fioretto P., Bruseghin M., Berto I., Gallina P., Manzato E. and Mussap M. (2006) Renal protection in diabetes: role of glycemic control. J. Am. Soc. Nephrol. 17, S86–S89 10.1681/ASN.2005121343 16565255

[8] Futrakul N. and Futrakul P. (2012) Therapeutic resistance to ACEI and ARB combination in macroalbuminuric diabetic nephropathy. Clin. Nephrol. 78, 250 10.5414/CN107538 22541688

[9] Sternlicht H. and Bakris G.L. (2016) Management of hypertension in diabetic nephropathy: how low should we go? Blood Purif. 41, 139–143 10.1159/000441264 26766168

[10] Chandra D., Jackson E.B., Ramana K.V., Kelley R., Srivastava S.K. and Bhatnagar A. (2002) Nitric oxide prevents aldose reductase activation and sorbitol accumulation during diabetes. Diabetes 51, 3095–3101 10.2337/diabetes.51.10.3095 12351453

[11] Taneda S., Honda K., Tomidokoro K., Uto K., Nitta K. and Oda H. (2010) Eicosapentaenoic acid restores diabetic tubular injury through regulating oxidative stress and mitochondrial apoptosis. Am. J. Physiol. Renal. Physiol. 299, F1451–F1461 10.1152/ajprenal.00637.2009 20844021

[12] Ha H., Yu M.R., Choi Y.J., Kitamura M. and Lee H.B. (2002) Role of high glucose-induced nuclear factor-kappaB activation in monocyte chemoattractant protein-1 expression by mesangial cells. J. Am. Soc. Nephrol. 13, 894–902 11912248 10.1681/ASN.V134894

[13] Gruden G., Setti G., Hayward A., Sugden D., Duggan S., Burt D. (2005) Mechanical stretch induces monocyte chemoattractant activity via an NF-kappaB-dependent monocyte chemoattractant protein-1-mediated pathway in human mesangial cells: inhibition by rosiglitazone. J. Am. Soc. Nephrol. 16, 688–696 10.1681/ASN.2004030251 15677312

[14] Kolati S.R., Kasala E.R., Bodduluru L.N., Mahareddy J.R., Uppulapu S.K., Gogoi R. (2015) BAY 11-7082 ameliorates diabetic nephropathy by attenuating hyperglycemia-mediated oxidative stress and renal inflammation via NF-kappaB pathway. Environ. Toxicol. Pharmacol. 39, 690–699 10.1016/j.etap.2015.01.019 25704036

[15] Kavitha K., Vidya P.R., Anitha P., Ramalingam K., Sakthivel R., Purushothaman G. (2012) Nimbolide, a neem limonoid abrogates canonical NF-kappaB and Wnt signaling to induce caspase-dependent apoptosis in human hepatocarcinoma (HepG2) cells. Eur. J. Pharmacol. 681, 6–14 10.1016/j.ejphar.2012.01.024 22327045

[16] Leibowitz B. and Yu J. (2010) Mitochondrial signaling in cell death via the Bcl-2 family. Cancer Biol. Ther. 9, 417–422 10.4161/cbt.9.6.11392 20190564 PMC2874116

[17] Nam M.S., Jung D.B., Seo K.H., Kim B.I., Kim J.H., Kim J.H. (2016) Apoptotic effect of sanggenol L via caspase activation and inhibition of NF-kappaB Signaling in ovarian cancer cells. Phytother. Res. 30, 90–96 10.1002/ptr.5505 26555861

[18] Xu Y., Yang B., Hu Y., Lu L., Lu X., Wang J. (2016) Wogonin prevents TLR4-NF-kappaB-medicated neuro-inflammation and improves retinal ganglion cells survival in retina after optic nerve crush. Oncotarget 7, 72503–72517 10.18632/oncotarget.12700 27756890 PMC5341925

[19] Bu Y., Li X., He Y., Huang C., Shen Y., Cao Y. (2016) A phosphomimetic mutant of RelA/p65 at Ser536 induces apoptosis and senescence: an implication for tumor-suppressive role of Ser536 phosphorylation. Int. J. Cancer 138, 1186–1198 10.1002/ijc.29852 26375985

[20] Wang J., Huang H., Liu P., Tang F., Qin J., Huang W. (2006) Inhibition of phosphorylation of p38 MAPK involved in the protection of nephropathy by emodin in diabetic rats. Eur. J. Pharmacol. 553, 297–303 10.1016/j.ejphar.2006.08.087 17074319

[21] Xiang L., Jiang P., Zhou L., Sun X., Bi J., Cui L. (2016) Additive effect of Qidan Dihuang Grain, a traditional Chinese medicine, and angiotensin receptor blockers on albuminuria levels in patients with diabetic nephropathy: A Randomized, Parallel-Controlled Trial. Evid. Based Complement Alternat. Med. 2016, 1064924 10.1155/2016/1064924 27375762 PMC4916306

[22] Lo H.Y., Li T.C., Yang T.Y., Li C.C., Chiang J.H., Hsiang C.Y. (2017) Hypoglycemic effects of Trichosanthes kirilowii and its protein constituent in diabetic mice: the involvement of insulin receptor pathway. BMC Complement. Altern. Med. 17, 53 10.1186/s12906-017-1578-6 28100206 PMC5242006

[23] Seo C.S., Kim T.W., Kim Y.J., Park S.R., Ha H., Shin H.K. (2015) Trichosanthes kirilowii ameliorates cisplatin-induced nephrotoxicity in both in vitro and in vivo. Nat. Prod. Res. 29, 554–557 10.1080/14786419.2014.952229 25185822

[24] Dat N.T., Jin X., Hong Y.S. and Lee J.J. (2010) An isoaurone and other constituents from Trichosanthes kirilowii seeds inhibit hypoxia-inducible factor-1 and nuclear factor-kappaB. J. Nat. Prod. 73, 1167–1169 10.1021/np900820p 20469887

[25] Li Q., Ye X.L., Zeng H., Chen X. and Li X.G. (2012) [Study on the extraction technology and hypoglycemic activity of lectin from Trichosanthes kirilowi]. Zhong Yao Cai 35, 475–479 22876690

[26] Yeung H.W., Ng T.B., Wong N.S. and Li W.W. (1987) Isolation and characterization of an abortifacient protein, momorcochin, from root tubers of Momordica cochinchinensis (family cucurbitaceae). Int. J. Pept. Protein Res. 30, 135–140 10.1111/j.1399-3011.1987.tb03321.x 3667075

[27] Ding X., Chi J., Yang X., Hao J., Liu C., Zhu C. (2017) Cucurbitacin B synergistically enhances the apoptosis-inducing effect of arsenic trioxide by inhibiting STAT3 phosphorylation in lymphoma Ramos cells. Leuk. Lymphoma 58, 2439–2451 10.1080/10428194.2017.1289521 28278714

[28] Malaisse W.J. (2006) Gliquidone contributes to improvement of type 2 diabetes mellitus management: a review of pharmacokinetic and clinical trial data. Drugs R.D. 7, 331–337 10.2165/00126839-200607060-00002 17073516

[29] Alaufi O.M., Noorwali A., Zahran F., Al-Abd A.M. and Al-Attas S. (2017) Cytotoxicity of thymoquinone alone or in combination with cisplatin (CDDP) against oral squamous cell carcinoma in vitro. Sci. Rep. 7, 13131 10.1038/s41598-017-13357-5 29030590 PMC5640598

[30] Yu C., Qi D., Sun J.F., Li P. and Fan H.Y. (2015) Rhein prevents endotoxin-induced acute kidney injury by inhibiting NF-kappaB activities. Sci. Rep. 5, 11822 10.1038/srep11822 26149595 PMC4493574

[31] Zhou L., Xu D.Y., Sha W.G., Shen L., Lu G.Y., Yin X. (2015) High glucose induces renal tubular epithelial injury via Sirt1/NF-kappaB/microR-29/Keap1 signal pathway. J. Transl. Med. 13, 352 10.1186/s12967-015-0710-y 26552447 PMC4640239

[32] Yang W.S., Chang J.W., Han N.J., Lee S.K. and Park S.K. (2012) Spleen tyrosine kinase mediates high glucose-induced transforming growth factor-beta1 up-regulation in proximal tubular epithelial cells. Exp. Cell Res. 318, 1867–1876 10.1016/j.yexcr.2012.05.016 22659134

[33] Lim A.K.H. and Tesch G.H. (2012) Inflammation in diabetic nephropathy. Mediators Inflamm. 2012, 146154 10.1155/2012/146154 22969168 PMC3432398

[34] Ren Y.X., Yang J., Sun R.M., Zhang L.J., Zhao L.F., Li B.Z. (2016) Viral IL-10 down-regulates the “MHC-I antigen processing operon” through the NF-kappaB signaling pathway in nasopharyngeal carcinoma cells. Cytotechnology 68, 2625–2636 10.1007/s10616-016-9987-9 27650182 PMC5101333

[35] Oguiza A., Recio C., Lazaro I., Mallavia B., Blanco J., Egido J. (2015) Peptide-based inhibition of IkappaB kinase/nuclear factor-kappaB pathway protects against diabetes-associated nephropathy and atherosclerosis in a mouse model of type 1 diabetes. Diabetologia 58, 1656–1667 10.1007/s00125-015-3596-6 25982245

[36] Hayden M.S. and Ghosh S. (2011) NF-kappaB in immunobiology. Cell Res. 21, 223–244 10.1038/cr.2011.13 21243012 PMC3193440

[37] Hinz M. and Scheidereit C. (2014) The IkappaB kinase complex in NF-kappaB regulation and beyond. EMBO Rep. 15, 46–61 10.1002/embr.201337983 24375677 PMC4303448

[38] Tang Q., Lu M., Zhou H., Chen D. and Liu L. (2017) Gambogic acid inhibits the growth of ovarian cancer tumors by regulating p65 activity. Oncol. Lett. 13, 384–388 10.3892/ol.2016.5433 28123571 PMC5244847

[39] Moheimani F., Tan J.T., Brown B.E., Heather A.K., van Reyk D.M. and Davies M.J. (2011) Effect of exposure of human monocyte-derived macrophages to high, versus normal, glucose on subsequent lipid accumulation from glycated and acetylated low-density lipoproteins. Exp. Diabetes Res. 2011, 851280 10.1155/2011/851280 21904540 PMC3166758

