# Supplementary material for: Trichosanthes kirilowii lectin alleviates diabetic nephropathy by inhibiting the LOX1/NF-κB/caspase-9 signaling pathway
Source: Biosci Rep. 2018 Sep 7;38(5):BSR20180071. doi: 10.1042/BSR20180071 (PMC6127671; doi:10.1042/BSR20180071)

Supplementary information

Table S1 The primer sequences used for real-time PCR assay in rats.

Gene	Primer (5' – 3')
IL-6	Forward: ATTGTATGAACAGCGATGATGCAC;<br>Reverse: CCAGGTAGAAACGGAACTCCAGA
Il-18	Forward: AGATAGGGTCACAGCCAGTC-3;<br>Reverse: GCTGCAATACCAGAAGAAGG
GAPDH	Forward: GAAAGACAACCAGGCCATCAG;<br>Reverse: TCATGAATGCATCCTTTTTTGC

Figure S1. TKL markedly decreased the blood glucose levels in STZ-induced nephropathy rat models.

(A) The blood glucose levels in rats of different groups before the treatments; (B) The blood glucose levels in rats of different groups after the treatments. Data are presented as mean ± SD; n=6, $^{\#\#}p<0.01$ versus control group; $^{*}p<0.05$ versus STZ group; $^{\&}p<0.05$ versus high-dose TKL group.

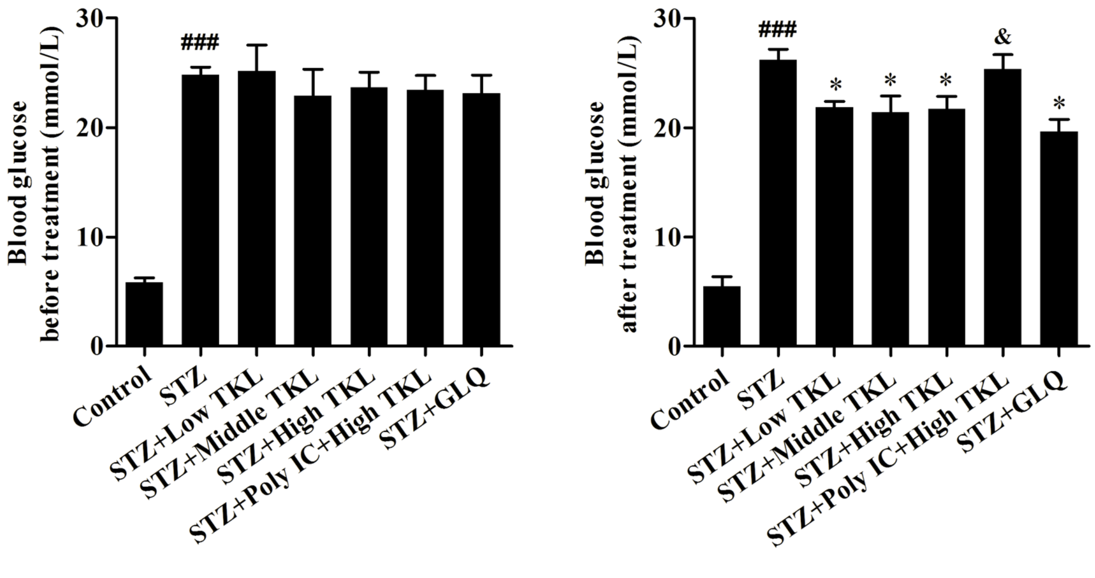

Supplement: Supplementary file 1 [file bsr20180071_Supp1.pdf]
